# Complete Genome Sequence and Methylome Analysis of Deinococcus wulumuqiensis 479

**DOI:** 10.1128/MRA.00066-19

**Published:** 2019-03-21

**Authors:** Alexey Fomenkov, Yvette Luyten, Tamas Vincze, Brian P. Anton, Richard J. Roberts, Richard D. Morgan

**Affiliations:** a New England Biolabs Inc., Ipswich, Massachusetts, USA; University of Maryland School of Medicine

## Abstract

Deinococcus wulumuqiensis 479 (formerly known as Deinococcus radiodurans 479) is the original source strain for the restriction enzyme DrdI. Its complete sequence and full methylome were determined using Pacific Biosciences single-molecule real-time (SMRT) sequencing.

## ANNOUNCEMENT

Deinococcus wulumuqiensis 479 was isolated in 1988 as part of a screening program for restriction enzymes having new specificities and is curated in the New England Biolabs culture collection (NEB 479). It is the original source of the prototype type II restriction enzyme DrdI that recognizes and cleaves the DNA sequence 5′-GACNNNN↓NNGTC-3′ (1). The genus *Deinococcus* is well known for extreme resistance to ionizing radiation, desiccation, and DNA-damaging chemicals (2).

Genomic DNA from an overnight L broth (3) liquid culture of Deinococcus wulumuqiensis 479 was purified using a modified protocol from a blood and cell culture DNA kit (Qiagen, USA) and sequenced using the Pacific Biosciences (PacBio) RS II sequencing platform. Briefly, SMRTbell libraries were constructed from a genomic DNA sample sheared to ∼10 to 20 kb using the g-tubes protocol (Covaris, Woburn, MA, USA), end repaired, and ligated to PacBio hairpin adapters. Incompletely formed SMRTbell templates and linear DNAs were digested with a combination of exonuclease III and exonuclease VII (New England Biolabs, Ipswich, MA, USA). DNA qualification and quantification were performed using the Qubit fluorimeter (Invitrogen, Eugene, OR) and 2100 Bioanalyzer (Agilent Technology, Santa Clara, CA). Two 15-kb SMRTbell libraries were prepared according to modified PacBio sample preparation protocols, including additional separation on BluePippin (Sage Science, Beverly, MA), originally sequenced using C2-P4 chemistry (7 SMRT cells, 120-min collection times) and later sequenced with C4-P6 chemistry (1 SMRT cell, 300-minute collection time). Sequencing reads (391,202 reads, mean subread length of 4,278 bp, and yield of 1.7 Gb for C2-P4; and 38,636 reads, mean subread length of 8,667 bp, and yield of 335 Mb for C4-P6) were assembled *de novo* using Hierarchical Genome Assembly Process (HGAP) assembly 1 version 2.1.1 (C2-P4 sequence) or HGAP assembly 3 version 2.3.0 (C4-P6 sequence) with default quality and read length parameters and polished using Quiver (4). Both assemblies gave 7 closed-circular genome elements with a G+C content of 65.62% (Table 1). The assembled sequence was annotated using the NCBI Prokaryotic Genome Annotation Pipeline (PGAP) (5, 6).

**TABLE 1:** Summary of genome elements, methyltransferase genes, and their motifs identified in Deinococcus wulumuqiensis 479

Genetic element	GenBank accession no.	Genome size (bp)	Genome coverage (×)	Methylase (RM system) name^*a*^	Recognition motif^*b*^	Methylation, RM type
Chromosome	CP031158	2,658,929	423.73	RM.DrdVIII^*c*^	ARG**A**GC	6mA, IIG
				RM.DrdV^*c*^	CATGN**A**C	6mA, IIG
				DrdIX	GNG**A**YNNNNNC****T****C	6mA, I
Plasmids						
pDrdI	CP031163	668,667	536.84	M.DrdI^*c*^	G**A**CNNNNNNG****T****C	6mA, II
				M.DrdORFCP	Not active	6mA, I
				M.DrdVII	Y**C**GC****G****R	5mC II
pDrdA	CP031159	311,711	511.45			
pDrdB	CP031160	70,951	678.58			
pDrdIV	CP031162	31,268	544.7	RM.DrdIV^*c*^	TACG**A**C	6mA, IIG
pDrdII	CP031161	21,536	544.9	RM.DrdII^*c*^	GAACC**A**	6mA, IIG
pDrdVI	CP031164	15,512	243.93	M1-2.DrdVI^*c*^	GC**A**GCC	6mA, IIG

a RM, restriction modification.

b Modified bases are in bold and bases opposite to them are bold and underlined.

c Methyltransferase (MTase) genes cloned and expressed in E. coli strain ER2796 (11). DrdIII activity has been previously reported (1). Recent analysis of this strain did not reveal any traces of DrdIII activity nor the presence of a CGATCG modified motif. The strain may have lost a plasmid encoding the DrdIII gene, or the enzyme called DrdIII may have come from a contaminant.

One advantage of the PacBio sequencing platform is its ability to detect the epigenetic state of sequenced DNA (7–9). Seven DNA methyltransferase recognition motifs were detected by single-molecule real-time (SMRT) motif and modification analysis version 2.1.1 and 2.3.0, each containing m6A modifications. While PacBio methylome analysis does not reliably detect m5C methylation, genome-wide m5C methylation analysis has been determined separately (B. P. Anton, unpublished data) to identify m5C recognition motifs. All motifs were matched with the responsible methyltransferases through cloning and expression in Escherichia coli, and the results are shown in Table 1 and have been deposited in REBASE (10).

### Data availability.

The complete genome sequence of Deinococcus wulumuqiensis 479 is available in GenBank under the accession numbers CP031158, CP031159, CP031160, CP031161, CP031162, CP031163, and CP031164. Original sequence reads have been deposited in NCBI under SRA accession number SRS4110296 and BioProject accession number PRJNA482107.
